# Proceedings of the Eighth Annual Deep Brain Stimulation Think Tank: Advances in Optogenetics, Ethical Issues Affecting DBS Research, Neuromodulatory Approaches for Depression, Adaptive Neurostimulation, and Emerging DBS Technologies

**DOI:** 10.3389/fnhum.2021.644593

**Published:** 2021-04-19

**Authors:** Vinata Vedam-Mai, Karl Deisseroth, James Giordano, Gabriel Lazaro-Munoz, Winston Chiong, Nanthia Suthana, Jean-Philippe Langevin, Jay Gill, Wayne Goodman, Nicole R. Provenza, Casey H. Halpern, Rajat S. Shivacharan, Tricia N. Cunningham, Sameer A. Sheth, Nader Pouratian, Katherine W. Scangos, Helen S. Mayberg, Andreas Horn, Kara A. Johnson, Christopher R. Butson, Ro’ee Gilron, Coralie de Hemptinne, Robert Wilt, Maria Yaroshinsky, Simon Little, Philip Starr, Greg Worrell, Prasad Shirvalkar, Edward Chang, Jens Volkmann, Muthuraman Muthuraman, Sergiu Groppa, Andrea A. Kühn, Luming Li, Matthew Johnson, Kevin J. Otto, Robert Raike, Steve Goetz, Chengyuan Wu, Peter Silburn, Binith Cheeran, Yagna J. Pathak, Mahsa Malekmohammadi, Aysegul Gunduz, Joshua K. Wong, Stephanie Cernera, Wei Hu, Aparna Wagle Shukla, Adolfo Ramirez-Zamora, Wissam Deeb, Addie Patterson, Kelly D. Foote, Michael S. Okun

**Affiliations:** ^1^Norman Fixel Institute for Neurological Diseases and the Program for Movement Disorders and Neurorestoration, Department of Neurology, University of Florida, Gainesville, FL, United States; ^2^Department of Bioengineering, Stanford University, Stanford, CA, United States; ^3^Department of Psychiatry and Behavioral Sciences, Stanford University, Stanford, CA, United States; ^4^Department of Neurology and Neuroethics Studies Program, Georgetown University Medical Center, Washington, DC, United States; ^5^Center for Medical Ethics and Health Policy, Baylor College of Medicine, Houston, TX, United States; ^6^Weill Institute for Neurosciences, Memory and Aging Center, University of California, San Francisco, San Francisco, CA, United States; ^7^Department of Neurosurgery, David Geffen School of Medicine and Semel Institute for Neuroscience and Human Behavior, University of California, Los Angeles, Los Angeles, CA, United States; ^8^Department of Psychiatry and Biobehavioral Sciences, Semel Institute for Neuroscience and Human Behavior, University of California, Los Angeles, Los Angeles, CA, United States; ^9^Department of Psychology, University of California, Los Angeles, Los Angeles, CA, United States; ^10^Department of Bioengineering, University of California, Los Angeles, Los Angeles, CA, United States; ^11^Neurosurgery Service, Department of Veterans Affairs Greater Los Angeles Healthcare System, Los Angeles, CA, United States; ^12^Menninger Department of Psychiatry and Behavioral Sciences, Baylor College of Medicine, Houston, TX, United States; ^13^School of Engineering, Brown University, Providence, RI, United States; ^14^Department of Neurosurgery, Stanford University Medical Center, Stanford, CA, United States; ^15^Department of Neurological Surgery, Baylor College of Medicine, Houston, TX, United States; ^16^Department of Psychiatry, University of California, San Francisco, San Francisco, CA, United States; ^17^Department of Neurology and Department of Neurosurgery, Icahn School of Medicine at Mount Sinai, New York, NY, United States; ^18^Movement Disorders & Neuromodulation Unit, Department for Neurology, Charité – University Medicine Berlin, Berlin, Germany; ^19^Department of Biomedical Engineering, University of Utah, Salt Lake City, UT, United States; ^20^Scientific Computing and Imaging Institute, University of Utah, Salt Lake City, UT, United States; ^21^Department of Neurological Surgery, Kavli Institute for Fundamental Neuroscience, University of California, San Francisco, San Francisco, CA, United States; ^22^Department of Neurology, Mayo Clinic, Rochester, MN, United States; ^23^Department of Anesthesiology (Pain Management) and Neurology, University of California, San Francisco, San Francisco, CA, United States; ^24^Neurologischen Klinik Universitätsklinikum Würzburg, Würzburg, Germany; ^25^Section of Movement Disorders and Neurostimulation, Biomedical Statistics and Multimodal Signal Processing Unit, Department of Neurology, Focus Program Translational Neuroscience, University Medical Center of the Johannes Gutenberg-University Mainz, Mainz, Germany; ^26^Department of Neurology, Charité – Universitätsmedizin Berlin, Berlin, Germany; ^27^National Engineering Laboratory for Neuromodulation, School of Aerospace Engineering, Tsinghua University, Beijing, China; ^28^Department of Biomedical Engineering, University of Minnesota, Minneapolis, MN, United States; ^29^J. Crayton Pruitt Family Department of Biomedical Engineering, University of Florida, Gainesville, FL, United States; ^30^Restorative Therapies Group Implantables, Research and Core Technology, Medtronic, Minneapolis, MN, United States; ^31^Department of Neurological Surgery, Thomas Jefferson University Hospitals, Philadelphia, PA, United States; ^32^Asia Pacific Centre for Neuromodulation, Queensland Brain Institute, The University of Queensland, Brisbane, QLD, Australia; ^33^Neuromodulation Division, Abbott, Plano, TX, United States; ^34^Boston Scientific Neuromodulation, Valencia, CA, United States; ^35^Department of Neurology, University of Massachusetts, Worchester, MA, United States

**Keywords:** DBS (deep brain stimulation), neuroethics, optogenetics, novel hardware, adaptive DBS, neuroimaging

## Abstract

We estimate that 208,000 deep brain stimulation (DBS) devices have been implanted to address neurological and neuropsychiatric disorders worldwide. DBS Think Tank presenters pooled data and determined that DBS expanded in its scope and has been applied to multiple brain disorders in an effort to modulate neural circuitry. The DBS Think Tank was founded in 2012 providing a space where clinicians, engineers, researchers from industry and academia discuss current and emerging DBS technologies and logistical and ethical issues facing the field. The emphasis is on cutting edge research and collaboration aimed to advance the DBS field. The Eighth Annual DBS Think Tank was held virtually on September 1 and 2, 2020 (Zoom Video Communications) due to restrictions related to the COVID-19 pandemic. The meeting focused on advances in: (1) optogenetics as a tool for comprehending neurobiology of diseases and on optogenetically-inspired DBS, (2) cutting edge of emerging DBS technologies, (3) ethical issues affecting DBS research and access to care, (4) neuromodulatory approaches for depression, (5) advancing novel hardware, software and imaging methodologies, (6) use of neurophysiological signals in adaptive neurostimulation, and (7) use of more advanced technologies to improve DBS clinical outcomes. There were 178 attendees who participated in a DBS Think Tank survey, which revealed the expansion of DBS into several indications such as obesity, post-traumatic stress disorder, addiction and Alzheimer’s disease. This proceedings summarizes the advances discussed at the Eighth Annual DBS Think Tank.

## Introduction

The Eighth Annual deep brain stimulation (DBS) Think Tank meeting was held virtually on September 1 and 2, 2020 (Zoom Video Communications) due to restrictions related to the COVID-19 pandemic. The DBS Think Tank presenters pooled data and determined that DBS has expanded in its scope and has been applied to multiple brain disorders. There have now been an estimated 208,000 DBS devices implanted for neurological and neuropsychiatric disorders worldwide. The DBS Think Tank was founded in 2012 and provides a space where clinicians, engineers, clinical-researchers, basic researchers and scientists from both industry and academia engage in discussions on current and emerging DBS technologies as well as tackle logistical and ethical issues facing the field. The DBS Think Tank has an emphasis on cutting edge research and collaboration which is aimed to more rapidly advance the DBS field.

The DBS Think Tank meeting was focused on advances in the following areas:

(1) optogenetics as a tool for comprehending the neurobiology of diseases and on optogenetically inspired DBS,
(2) the cutting edge of emerging DBS technologies,
(3) ethical issues affecting DBS research and access to care,
(4) neuromodulatory approaches for depression,
(5) advancing novel hardware, software and imaging methodologies,
(6) the use of neurophysiological signals in adaptive neurostimulation,
(7) the use of more advanced technologies to improve DBS clinical outcomes,
(8) the use of novel techniques such as INTRSECT (intronic recombinase sites enabling combinatorial targeting),
(9) an updated survey of 178 attendees which is performed each year to track trends in the field.

These proceedings will summarize the Eighth Annual DBS Think Tank meeting.

## Optogentically-Inspired DBS

Optogenetics has advanced our comprehension of the pathophysiology and neurobiology of disease, and continues to bring promise to our fundamental comprehension of the role of specific cell types, and even single cells, in the brain. Channelrhodopsins are naturally occurring light-gated ion channels in algae, which have become important in neuroscience research for targeted control of specific circuit elements with optogenetic techniques. Here we discuss how optogenetics as a research tool can be used to uncover underlying circuitry and to motivate new approaches for applying DBS into the human population.

### Inner Workings of Channelrhodopsins

It was Francis Crick who first suggested the rather far-fetched idea that light could be a useful tool for the investigation of neural function and that it could be used in a targeted manner. Since then, converging advances in genetics, optics and engineering have collectively shown substantial promise for the investigation of neurological diseases. Current neuromodulation methods (achieved via DBS) tend to stimulate all neurons in a certain volume of tissue, which may include cells not involved in disease and thus likely result in undesirable side-effects. Hence, controlling the activity of specific neurons has significant potential to advance the field of neuromodulation. Cell specific excitation (or inhibition) of neurons can be achieved using light via microbial opsins, which encode all-in-one proteins including ion channels called channelrhodopsins that transduce photons into electrical current (Nagel et al., 2002; Deisseroth and Hegemann, 2017), enabling the first temporally precise control of genetically targeted cells in behaving mammals (Adamantidis et al., 2007). One of the early studies showing the generality of this methodology in an exploration of the role of bed nucleus of the stria terminalis (BNST) circuit elements in modulating anxiety, involved integrating behavior, electrophysiology, respiratory physiology, and optogenetics. It was discovered that three BNST efferent projections—to the lateral hypothalamus, parabrachial nucleus, and ventral tegmental area—each corresponded to a unique aspect of anxiolysis: that is, reduced risk-avoidance, reduced respiratory rate, and positive valence of the state, respectively (Kim et al., 2013). Subsequent studies revealed that optogenetics recruits naturalistic patterns of downstream neuronal population activity (Allen et al., 2019; Jennings et al., 2019). One of these studies (Allen et al., 2019) utilized optogenetics to recruit neurons that were normally activated upon deprivation of water, by providing input to the median preoptic nucleus of the hypothalamus (MnPO), while simultaneously recording from thousands of neurons across the brain using electrophysiology; all during behavior. It was found that targeted optogenetics recruits a naturalistic brain-wide pattern of activity like that elicited by natural thirst and water-seeking behavior (Allen et al., 2019).

Recruitment of opsins to modulate neuronal circuitry at the single cell level in living mammals was initially achieved via two photon activation of neurons (Prakash et al., 2012). This ultimately enabled the first specified-single-cell control of mammalian behavior, via interrogation of orbitofrontal (OFC) neurons during distinct and different behaviors: feeding responses and social interaction (Jennings et al., 2019). Feeding responsive OFC neurons were selected for optogenetic control; it was found that specific modulation of these cells was able to enhance feeding behavior. In order to determine if these behavioral effects associated with stimulation were specific to the feeding cells, OFC cells not involved in feeding behavior (social behavior-responsive cells) were stimulated, and found to instead result in inhibition of feeding. These experiments inform on the role of well-defined OFC networks involved in feeding and social behaviors and demonstrate that mammalian behavior can be specifically controlled via modulation of individual cells within a network, and that optogenetic identification of subnetworks results in elucidation of the dynamics involved in primary motivational drives. The discovery of ChRmine, a fast and highly sensitive red-shifted opsin, has recently facilitated precise control over large ensembles of individually specified single cells in behavior (Marshel et al., 2019), and is suitable for deep transcranial optogenetic modulation of behavior in mouse, as was seen in a later paper addressing multiple specific behaviors including appetitive conditioning (Chen R. et al., 2020). This methodology makes it possible for the exploration of therapeutic interventions wherein the source of light can be distinctly separated from the target cell population. Thus, specific adaptive behavior can be elicited via deep transcranial ChRmine photoactivation, which precludes the need for intracranial surgery. In parallel with the revolution arising from optogenetic approaches, the recent explosion of single-cell transcriptomic data has made clear that cell types can be usefully targeted by more than one genetic feature. INTRSECT (intronic recombinase sites enabling combinatorial targeting) addresses this opportunity by allowing the expression of adeno associated virus (AAV) based payloads by combining synthetic introns, and two recombinases (Cre and Flp) defining cellular populations specified by two features. INTRSECT has been used to identify functional roles for projection patterns of diverse neuronal subtypes (Chuhma et al., 2018; Poulin et al., 2018; Fenno et al., 2020) in physiology and behavior. Further development of this approach has resulted in a triple recombinase dependent gene targeting approach (Triplesect) (Fenno et al., 2020); this technology can achieve superior viral targeting specificity and will likely result in the ability to study the role of cell types that are triply defined genetically and/or anatomically.

## Advances in Commercially Available Neuromodulation Technologies

Over the past years, chronically sensed brain signals have been established as an important new opportunity for advancing the standard of care (SOC) in DBS therapies. Historically, access to such signals has been available on a limited basis via investigational devices specialized for such recordings, which have collectively allowed constrained exploration of such signals in research contexts. However, these signals were the impetus of significant scientific and technological discoveries in the DBS space. In the research literature, it has been shown that such signals are robust and chronically present over months to years (Abosch et al., 2012; Giannicola et al., 2012; Trager et al., 2016; Neumann et al., 2017), that they often correlate with patient symptoms and additionally can be used with the delivery of therapy (both stimulation and medications) (Kuhn et al., 2006; Quinn et al., 2015; Trager et al., 2016; Neumann et al., 2017), and finally that it is feasible and there may be benefits to applying closed-loop methodologies using these correlations to adapt therapy over time and thereby adjust for fluctuations in symptoms (Velisar et al., 2019; Petrucci et al., 2020). With the first availability of commercial devices (Percept PC^TM^) to implement chronically sensed brain signals, these opportunities for clinical value have become available more broadly. Very early evidence (Koeglsperger et al., 2020) suggests that the findings of the research community can be replicated in these commercial devices, demonstrating the feasibility of using in-clinic signals during the programming process and at-home signals to understand the real-world characteristics of signals outside the clinic. The next opportunity to be explored is chronic closed-loop or adaptive therapies in naturalistic settings. Medtronic’s Percept PC^TM^ is enabled for these types of control algorithms through a software unlock, and these capabilities will be explored via industry-sponsored studies in Parkinson’s disease (PD; e.g., ADAPT PD – NCT04547712) beginning early in 2021. The Percept PC^TM^ platform has also been architected to unlock other advanced capabilities with appropriate regulatory approvals for research, including novel stimulation waveforms, network connectivity, and directional sensing with DBS leads bearing segmented electrodes. Research-enabled commercial device platforms such as the Percept PC^TM^ are poised to enable more rapid research translation through faster access to technological innovations and also offer fewer tradeoffs to clinical researchers and to research subjects.

Advancement of DBS device technology has made it possible to have multiple combinations of DBS programming settings in an effort to deliver better outcomes. Device programming on the other hand has only become more complicated. The development of a computer-guided closed-loop based programming algorithm could potentially make DBS programming easier for clinicians. In this context, Boston Scientific Neuromodulation (BSN) is working to improve tools to aid the clinical DBS workflow. These tools broadly include computer-aided programming (using objective outcome measurements), as well as stimulation field modeling with specificity to patient anatomy, which has been pursued through Boston Scientific’s CLOVER Study. CLOVER (NCT03037398) is a multi-center study which uses direct and objective symptom measures, such as from PD-validated, commercial finger-worn accelerometers, and integrates these measurements with a BSN-developed search and optimization algorithm. After three starting measurements, this algorithm iteratively suggests the next settings to test, until an optimum setting is found. Such an algorithm could assist both in-clinic and in remote programmers. BSN has recently updated the CLOVER algorithm to support programming of their directional leads. The preliminary results indicate that the new algorithm is able to converge in a single visit on stimulation settings that result in UPDRS motor score reductions (as compared to the baseline scores) that are statistically equivalent to multi-visit SOC programming (as defined by the clinician in the study) (Sasaki et al., 2021).

Programming may also be aided using patient imaging data paired with three-dimensional stimulation models in the Guide XT software, developed in collaboration with BrainLab. When available, the combination of surgical, imaging, and stimulation response (such as aggregated therapy sweet spots) with real-time clinical response may further assist DBS programmers. BSN is working toward tools to enable large scale, group studies to further investigate the relationships between stimulation locations and clinical outcomes, including using population statistics in order to build probabilistic maps of stimulation. The results of these population-based analyses could be used to inform programming software. More research is needed to further explore the predictive value of these maps and their potential use in routine clinical DBS programming practices.

Real-world analyses of claims data have previously shown a higher rate of DBS revision/removal procedures than typically recognized (Rolston et al., 2016). The impact of modern systems, with significant advancements in design and manufacturing, has not been previously studied. Abbott labs presented a study evaluating the impact of a modern DBS system on revision and replacement rates. Medicare fee for service claims were used to identify patients undergoing DBS implantation for PD or Essential Tremor between January 1, 2016–December 31, 2018. Claims records were linked to manufacturer device registration data to identify which patients had been implanted with Abbott Infinity, at the time the only commercially available system in the United States with directional stimulation capability; linked patients were assigned to the Treatment (Directional System) group. A total of 3,271 patients in Omnidirectional and 596 patients in Directional System group met the inclusion/exclusion criterion. Revision or replacement rates in patients implanted with the Infinity directional DBS system were significantly reduced compared to those with traditional omni-directional DBS systems. Further analysis and future studies may elucidate the mechanism of reduced risk. Another study evaluated an investigational software extension that enabled remote programming of previously implanted DBS devices. The paucity of trained neurologists and urban concentration of specialty care centers has contributed to care access burden for patients with DBS and their caregivers, particularly in the context of the COVID-19 pandemic. Remote monitoring^1^ and remote support technologies^2^ have been established in other Class 3 active implantable technologies. Remote programming of DBS systems has been enabled by China-based manufacturers but is not yet available in countries requiring CE or FDA approvals (Zhang et al., 2018). Abbott investigated an investigational remote programming feature to enable programmers to directly adjust DBS therapy settings in real-time via a secure video-based mobile platform in patients implanted with an Infinity^TM^ DBS system^3^. The primary endpoint of this study was to determine remote programming safety by evaluating adverse events (AEs) reported by subjects within three weeks of a programming session which was conducted using the remote programming feature. Ten subjects connected with their treating clinician through the secure remote programming feature. No serious AEs were reported in this study and anticipated, non-serious AEs that were reported for 1 subject resolved without sequalae. Evaluation of such remote programming features will likely advance the field towards low-burden therapy options for patients and clinicians in the rapidly emerging digital health realm.

## On Target, and (Yet) Off-Label Uses Of DBS: Ethical Concerns, Caveats, and Considerations

With the increased investigation and subsequent use of novel DBS therapies, there has been a simultaneous growth in ethical issues and considerations. One important issue that has been emerging is continued device access after the conclusion of a research study, which is generally considered to be ethically appropriate and desirable. Ascertaining whether researchers and industry sponsors are ethically obligated to facilitate continued access to those participants whose benefit requires a dialogue and engagement of all the relevant stakeholders. Additionally, the potential of DBS technologies for off-label use and the ethics surrounding this, specifically in vulnerable patient populations, must also be developed and subsequently reviewed.

### DBS for Less Prevalent Diseases, Continued Access After Trials and the NIH BRAIN Initiative Ethics Updates

The use of various forms of neurotechnologies and techniques to define and model loci for possible interventional neuromodulation is opening new vistas of “on target,” and (yet still) off-label uses of DBS (Ramirez-Zamora et al., 2017, 2019) see, for example, this report. As we have noted, such new horizons of possibility must be approached with ethical probity (Giordano, 2015). The novelty of utilizing DBS in such ways mandates explication of current uncertainties about the durability of clinical benefit, future side effects, and sustainability of intervention as contingencies for informed patient consent (Giordano, 2016).

That DBS affords effective clinical benefit can be seen as only an initial component (and hurdle) of successful care. Indeed, there have been—and remain—ethical and policy challenges regarding post-trial management of brain implant devices (Lazaro-Munoz et al., 2018; Sierra-Mercado et al., 2019). Brain implant trials generally do not have provisions to ensure that patients/subjects who gain clinical benefit from the use of DBS will have access to maintenance of the device after completion of the trials.

Patients who participate in these trials have severe and treatment-resistant neuropsychiatric conditions. Axiomatically, patients with “treatment-resistant” disorders who benefit from an experimental DBS intervention during a trial have no other effective treatment alternatives; and guaranteed provision of services and resources to assure maintenance of DBS devices upon completion of such trials is lacking. To be sure, such continued maintenance may incur significant costs. While sustainability of these devices for extant (CPT-code listed) indications *may* be covered by health insurance providers, such coverage is not obligated (and therefore is routinely not provided) for those indications that are experimental (Rossi et al., 2017). The significant burdens (i.e., surgery, multiple clinical visits) incurred by participants in these trials heighten their dependence upon the study teams for access to the only intervention that has afforded them successful clinical outcomes. Thus, we posit that these patients’ vulnerability is increased, and in this light, strongly advocate development of a system to ensure (and *insure*) post-trial continuity of clinical care for those patients/subjects for whom therapeutic benefits are achieved. Public and private research sponsors, device manufacturers, researchers—and the institutions in which this research occurs—can, and we believe should, play active roles in facilitating both the discourse as well as the resources required to assure these patients’ continued access to successful clinical care.

Of additional interest, particularly for experimental (i.e., off-label) uses of DBS are potential side effects that can occur in either the short, intermediate, or long-term. Provocative questions have arisen whether closed-loop neuromodulation could induce changes to a patients’ sense of identity and agency. Ongoing discussions in the literature have been equivocal, noting a paucity of data and arguing that such concerns may be overdrawn (Giordano, 2016). To address the need for empirical investigations, we at UCSF examined constructs and the subjective experience of identity in patients with refractory epilepsy undergoing responsive neurostimulation (RNS)—the first FDA-approved, commercially available closed-loop brain stimulation system. Pre- and post-implantation observations of 12 patients were conducted, in addition to in-depth interviews with both these patients and their respective caregivers. These interviews revealed that patients and caregivers did not attribute any perceived changes in patients’ identity or agency to the device’s operation, thereby refuting concerns that have been raised in the (conceptual) neuroethics literature. When such changes were noted, they were readily and characteristically described by patients and caregivers as attributable to their disorder, or as side effects of medications. Importantly, these reports indicated that the qualitative techniques used were able to elicit such concerns if and when present.

An unexpected finding was that the ability to view the neural recordings collected by the device was regarded as highly meaningful and personally significant to patients and caregivers; in some cases, independent of the device’s stimulation algorithm and/or effect(s). Notably, patients reported that neural recordings enabled visual demonstration of the disease process in ways that affected their understanding of the disorder, and themselves. These are the first such empirically obtained findings from clinical populations undergoing closed-loop neuromodulation, which we believe illustrate—and support—how empirical studies can and should inform the conceptual neuroethics literature.

## Depression DBS: Where Can We Go? Less vs More for DBS Depression

Major depressive disorder (MDD) is a prevalent disease, and one of the leading causes of disability worldwide (Giacobbe et al., 2009). A failure to identify and treat depression can have profound negative public health impacts such as hospitalizations, inter-personal issues, lack of productivity, and suicide. After early randomized controlled trials failed to show improvement, it is now becoming increasingly evident that DBS can be useful for treatment resistant depression, and several studies are showing promise (Holtzheimer et al., 2017; Hitti et al., 2020; van der Wal et al., 2020) Bergfeld, 2020). Stimulation of the subgenual cingulate has been shown to produce clinical benefits in patients with treatment resistant depression (Mayberg et al., 2005). Increased clinical benefits in these trials has stemmed from improvements in neuroimaging, personalized targeting, neurophysiology and stimulation delivery. Neuroimaging has aided in personalized lead targeting by defining critical white matter tracts that may be crucial in the pathology of depression. Furthermore, sites (discussed herein) have been using a network-based approach adopted from epilepsy which entails the temporary implantation of stereo-EEG electrodes either to study the network involved in depression, to choose optimal stimulation settings, or to demonstrate biomarkers that can be used in a closed-loop paradigm. As these neurophysiologic data are collected in both the temporary and long-term settings using devices such as the Summit RC + S, the heterogeneity in response to DBS may be elucidated and more refined symptom-specific biomarkers may be discovered. These advances will ultimately produce optimized DBS paradigms which are specific to each patient’s symptoms. Here we describe advances made in the field of DBS for depression across three different centers.

### Baylor Preliminary Experience Depression DBS Trial

**FIGURE 1:**
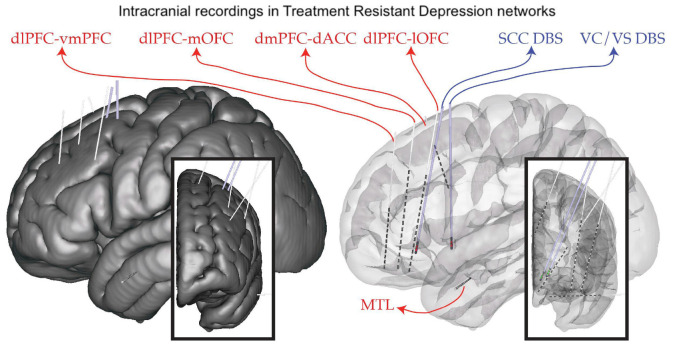
Implant plan. StereoEEG electrodes (red) are placed in a variety of brain regions thought to be part of the depression network (dorsolateral prefrontal cortex, dlPFC; ventrolateral PFC, vlPFC; dorsomedial PFC, dmPFC; medial and lateral orbitofrontal cortex, mOFC, lOFC; dorsal anterior cingulate cortex, dACC; medial temporal lobe, MTL). DBS leads (blue) are placed in the subcallosal cingulate (SCC) and ventral capsule/ventral striatum (VC/VS). Placement is individualized using tractography derived from diffusion MRI.

Deep brain stimulation for severe, treatment-resistant depression (TRD) is an investigational therapy. Previous studies have shown heterogeneous results, with early open-label studies demonstrating promise (Holtzheimer et al., 2012); however, industry-sponsored, blinded randomized trials were stopped at interim analyses points without demonstrating a difference between active and sham stimulation (Holtzheimer et al., 2012). We propose that an important limitation in applying DBS for this indication has been an incomplete understanding of the network of brain regions responsible for the multifactorial dysfunction underlying depression. To address this limitation, we applied an approach borrowed from another challenging and highly individualized disorder: epilepsy. As is done commonly in epilepsy, our study involves intracranial recordings using temporarily placed stereo-EEG (sEEG) electrodes in brain regions hypothesized to be within the TRD networks. We simultaneously place permanent DBS leads in two bilateral regions: the ventral capsule/ventral striatum (VCVS) and sub-callosal cingulate (SCC) regions (Figure 1). The patient is kept in the hospital “neurophysiological monitoring unit” (NMU) for 10 days and undergoes a number of recording and stimulation activities to understand brain network neurophysiology across a variety of states (resting/baseline, emotional valence states, cognitive effort states) and in response to stimulation across a variety of stimulation parameters (frequency, pulse width, amplitude, direction). One of the many goals of this intracranial recording phase is to narrow the vast parameter space to a few parameter sets that can be implemented in the chronic outpatient phase of the trial.

We report the results from the first patient in this trial (NCT03437928), which is funded by the NIH BRAIN Initiative (UH3 NS103549; PIs Sheth, Pouratian, Goodman). This trial was approved by the FDA (IDE G180300) and IRB. We gathered a plethora of data during the intracranial phase that helped to create a model of the relationship between imaging, neurophysiology, and behavioral/symptomatic response. We used this information to create three parameter sets, which we tested in the outpatient phase. Implementing these individualized parameter sets across the four DBS leads led to a steady reduction in symptom scores, such that the subject achieved symptom remission by 22 weeks.

We propose that “sEEG-guided DBS” is a useful platform for developing a network understanding of disorders and that this approach will provide sufficient information to optimize neuromodulatory therapies such as DBS. Future challenges include balancing the competing drives of optimizing previously studied DBS targets versus exploring new targets, properly interpreting acute results for chronic use, and translating this paradigm so that in the future, inpatient intracranial recordings will not be needed.

### UCSF Preliminary Experience From an Ongoing Depression Trial

MDD is a common and highly disabling disorder worldwide. While the majority of patients respond well to medication and psychotherapy, a substantial number of patients remain refractory to all available treatments. DBS is a highly promising therapy for this subset of patients with treatment resistant disease. However, results from randomized controlled studies of DBS for depression have not been consistent, suggesting that novel strategies in DBS treatment are needed. Three approaches toward DBS optimization in depression are currently underway by groups at Mt. Sinai, Baylor and UCSF. They include enhanced target engagement through tractography and biomarker development, DBS parameter optimization through individualized network targeting, and development of a personalized closed-loop paradigm.

**FIGURE 2:**
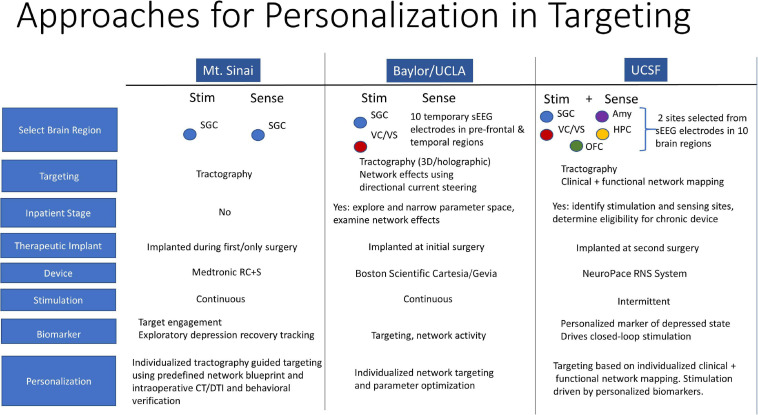
Personalization in Targeting. Closed-loop DBS model for depression, patient selection, and personalized clinical mapping integrating clinical responses, functional and structural connectivity mapping.

At UCSF, we are conducting a 3-stage feasibility study of personalized closed-loop stimulation for treatment resistant MDD. Surgical implantation of 10 intracranial EEG electrodes allows for personalized stimulation site selection and biomarker discovery over 10 days of intensive in-patient monitoring. Intracranial-EEG electrodes are then removed and a chronic DBS device (NeuroPace RNS^®^ System) is implanted in sensing and stimulation targets identified in the discovery stage. An open label period follows where a biomarker-based detection algorithm is developed and integrated into closed-loop therapy and then tested through a randomized controlled study. In this talk, we discuss the rationale behind a closed-loop DBS approach. We discuss the conceptualization of depression in a closed-loop model, implications for patient selection, and a strategy for personalized clinical mapping that integrates clinical responses with functional and structural connectivity mapping. We highlight differences of our approach in comparison to the approaches at Mt. Sinai and Baylor and suggest strategies for integration of the three complementary efforts (Figure 2).

### Optimizing SCC DBS for TRD Using Chronic Sensing: Less Versus More

**FIGURE 3:**
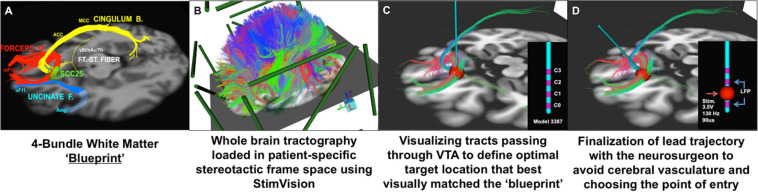
Individualized tractography-guided, template-matching lead implantation procedure for SCC DBS for TRD. **(A)** 4-bundle tractography target template (Riva-Posse et al., 2014). **(B)** Overlap of whole-brain deterministic tractography in patient-specific stereotactic frame space using the “StimVision” toolbox (Noecker et al., 2018). **(C)** Initial placement of electrode within SCC and visualization of WM pathways passing through the VTA. **(D)** Personalized optimal electrode location with the arc and ring angle determination by a neurosurgeon. Estimated VTA with standard stimulation settings (i.e., 3.5V, 130Hz, 90ms) is visualized with local field potential recording from adjacent contacts (sandwiching recording to minimize stimulation artifact). Composite images courtesy of Ki Sueng Choi, Icahn School of Medicine at Mount Sinai.

It has been 15 years since the first proof-of-principle report of DBS for treatment resistant depression, targeting the subcallosal cingulate (SCC) region (Mayberg et al., 2005). Initial studies were catalyzed by critical clinical need, informed by converging findings from imaging studies of depression pathophysiology and antidepressant treatment, and operationalized using established imaging standards for movement disorder surgery including trial-and-error behavior testing during chronic stimulation at individual contacts on each implanted DBS lead (Kennedy et al., 2011; Holtzheimer et al., 2012). As SCC DBS has evolved and matured, neuroimaging continues to play a crucial role, with implementation of refined multimodal techniques for surgical targeting and long-term studies of treatment mechanisms (Crowell et al., 2019). Most critically, increased precision has been achieved with implementation of an individualized tractography-guided, template-matching lead implantation procedure (Figure 3), now successfully deployed in two successive cohorts, with a resulting 6- month response rate of 80% (8 of 11 patients) (Riva-Posse et al., 2018) and 90% (9 of 10 patients) (unpublished), respectively.

This standardized method for reproducible lead implantation and contact selection for chronic stimulation has been further verified by robust and reproducible intraoperative behavioral effects associated with unilateral and bilateral therapeutic stimulation at the predefined targets (Smart et al., 2018; Riva-Posse et al., 2020). With this critical variable of reliable targeting now achieved, current experiments have been focused on outpatient strategies to characterize the unexplained differences in the speed and trajectory of antidepressant effects facilitated by chronic DBS. Such studies have been enabled by device innovations, specifically the Activa PC + S and Summit RC + S systems (Medtronic), that have facilitated ongoing interrogation of DBS mechanisms at the neural level (Veerakumar et al., 2019). To further refine and optimize DBS treatment, neural biomarkers that reliably track the depression state over time and that can discriminate depression relapse from transient fluctuations in negative mood and from arousal are needed. Ideally, these new brain tracking metrics would be derived using novel quantitative behavioral assessments that are not totally dependent on patient self-report. While standardized depression rating scales are generally effective in establishing the clinical efficacy of DBS, they may be inadequate to develop neural control policies in order to monitor and to optimize DBS delivery.

To this point, preliminary studies have demonstrated that following 2 months of therapeutic SCC DBS, machine learning models of facial expression and vocal inflection drawn from unstructured patient interviews can reliably predict 6-month outcomes, outperforming classical depression severity rating scales (Harati et al., 2020). However, these methods have not yet fully exploited the richness of the available recorded brain derived datasets. Quantitative analyses of facial, voice, and body dynamics combined with concurrent home and lab recordings of SCC LFPs and self-paced video diaries have been undertaken (in progress) to test this hypothesis, with the goal to further streamline and to optimize SCC DBS for TRD.

## New Hardware/Software/Imaging

Advances in MRI technology has made it feasible to visualize brain networks in a manner previously thought to be impossible. The Human Connectome Project has enabled the generation of publicly available normative connectomes, which has proven invaluable for neuromodulation research (Yeo et al., 2011; Van Essen et al., 2012; Holmes et al., 2015). Neuroimaging improvements have enabled the identification of pathological circuits responsible for symptom manifestations, thus potentially leading to advances like personalized, connectivity-driven lead targeting. Furthermore, neuroimaging can help aid in the accurate mapping of the patient-specific stimulated area, lending to the understanding of either the improvement/worsening of symptoms or potential off-target side effects. Herein, we discuss recent advances in the use of imaging technology for improving DBS precision and outcomes.

### Toward Precision Imaging and Connectomic Surgery

**FIGURE 4:**
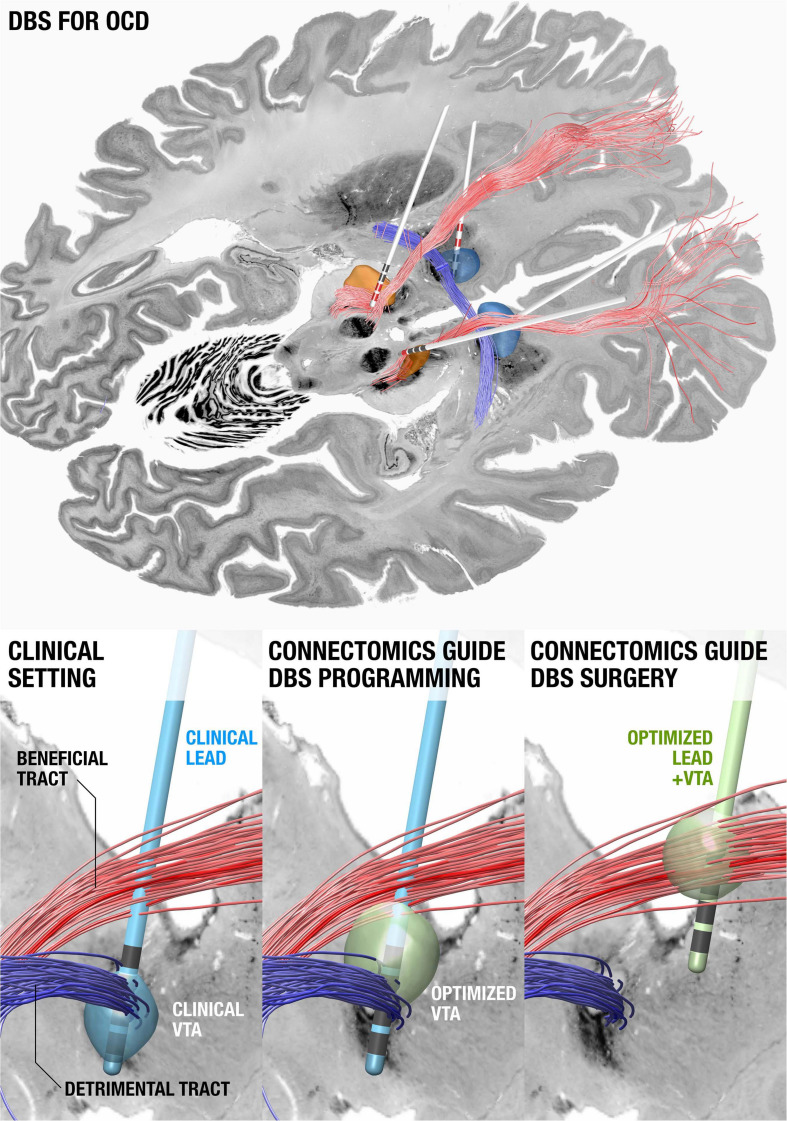
Using connectomics to guide surgery and DBS programming. **(Top)** DBS tract filtering. Four DBS electrodes implanted to the anteromedial subthalamic nucleus and anterior limb of the internal capsule in a patient with obsessive compulsive disorder. Active contacts are marked in red. A tract associated with optimal clinical improvement across 50 patients (limbic hyperdirect pathway within the anterior limb of the internal capsule) is shown in red, one associated with poor improvement (posterior limb of the anterior commissure) in blue. **(Bottom left)** Clinical DBS setting. **(Bottom middle)** Upon further confirmation of results, based on the existing electrode and the connectomic information, the stimulation settings could be optimized. **(Bottom right)** In novel patients, both surgical targeting and DBS programming could potentially be optimized. Data from Li et al. (2020), background slices show the BigBrain dataset (Amunts et al., 2013) after precise co-registration.

Shortly after publishing their seminal paper about a stereotaxic apparatus for human brain surgery in 1947 (Spiegel et al., 1947). Ernest Spiegel and Henry Wycis published the concept of *ansotomy* for treatment of Parkinsonian tremor (Spiegel and Wycis, 1954) with the aim to cut pallidofugal efferent fibers within the *ansa lenticularis* (Meyers, 1951). Hence, the concept of retuning brain function by modulating brain connectivity is actually a previously explored notion. What is new is our ability to integrate electrode localizations with connectome data non-invasively acquired using advanced MRI technology. Pioneered by multiple groups worldwide (Coenen et al., 2009, 2011; Anderson et al., 2011), this concept has become increasingly powerful in order to understand how the effects of DBS may impact the brain. When mapping DBS electrodes with neuroimaging, it is crucial to attain the highest degree of accuracy possible; since millimeters matter. Specialized neuroimaging pipelines that have this goal in mind include multispectral registration algorithms (Ewert et al., 2019), correction for bias introduced by brain shift (Horn et al., 2019), phantom-validated electrode reconstructions, and correction for detection of directionality in the specific case of segmented leads (Dembek et al., 2019). These tools allow us to precisely register DBS stimulation sites to other datasets, such as histological atlases (Ewert et al., 2018; Ilinsky et al., 2018), postmortem imaging (Edlow et al., 2019), or to normative connectome data aggregated from thousands of subjects (Horn et al., 2017a; Horn and Fox, 2020). In cases where patient-specific connectivity data is unavailable, normative connectome data can be an effective surrogate (Baldermann et al., 2019; Wang et al., 2020) and can potentially add the advantage of higher precision and increased signal-to-noise ratios when including data from the postmortem specimen (Calabrese et al., 2015), histology (Alho et al., 2020), or with integrated anatomical expert knowledge (Petersen et al., 2019; Middlebrooks et al., 2020; Figure 4).

A first report that applied this concept calculated a model of optimal connectivity in a cohort of PD patients and used these data to predict clinical improvement in a second cohort from a different center (Horn et al., 2017b). Since this publication, the concept has been applied to essential tremor, dystonia, and epilepsy (Middlebrooks et al., 2018; Al-Fatly et al., 2019; Okromelidze et al., 2020) and has been applied to predict side-effects. Using a related technique termed *tract-filtering*, specific bundles associated with optimal clinical improvement can be identified. This technique was first used in an obsessive compulsive disorder (OCD) cohort (Li et al., 2020).

In the future, a similar methodology could be used to define symptom-specific circuitopathies that may ultimately facilitate personalization of DBS targets to a somatotopic domain and to a symptom-spectrum of individual patients. Furthermore, advanced imaging technology will likely continue to enter the operation room in an effort to integrate surgical targets with a variety of neuroimaging data.

### Utilizing Multi-Country Imaging and Clinical Outcomes for Neuromodulation

Tourette syndrome (TS) is a complex neuropsychiatric disorder characterized by tics and often associated with psychiatric comorbidities, such as obsessive-compulsive behavior (OCB). DBS is an effective therapy for select patients with severe, treatment-refractory TS. However, patient responses to DBS are variable and there are currently no reliable predictors of symptom improvement. One contributing factor to the variability in clinical outcomes is the uncertainty into how to optimally target stimulation to improve tics or OCB. Progress toward identifying predictors of symptom improvement and effective neuroanatomical structures for stimulation has been limited by the relative paucity of TS cases implanted at individual centers. The International TS DBS Registry and Database (Deeb et al., 2016) was established to overcome this limitation by aggregating data from multiple international centers, including clinical data, stimulation settings, clinical rating scale scores, and pre- and postoperative imaging.

**FIGURE 5:**
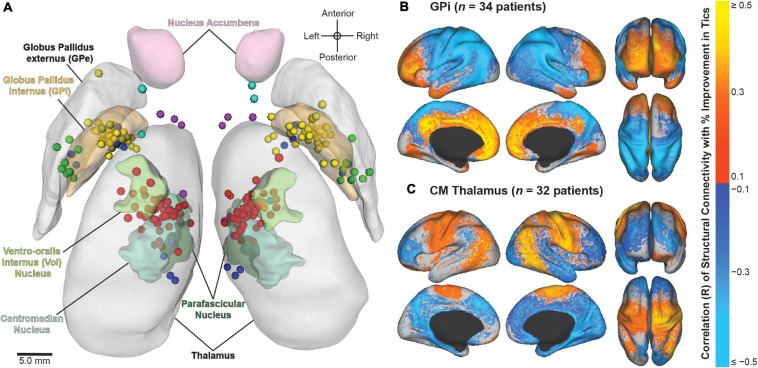
Image-based analyses of multicenter data from the International Tourette Syndrome (TS) Deep Brain Stimulation (DBS) Registry and Database. **(A)** Active contact locations for *N* = 70 patients implanted in the centromedial (CM) thalamus (red); anteromedial globus pallidus internus (GPi) (yellow); posteroventral GPi (green); nucleus accumbens/anterior limb of internal capsule (NA/ALIC) (turquoise); CM thalamus and GPi (blue); or CM thalamus and NA/ALIC (purple). From Johnson et al. (2019). **(B,C)** Stimulation-dependent structural connectivity associated with improvement in tics in patients implanted with DBS in the **(B)** GPi or **(C)** CM thalamus. From Johnson et al. (2020).

Using multicenter data from the registry, recent studies have aimed to identify the neuroanatomical structures associated with improvement in tics and comorbid OCB in patients who have undergone DBS for TS. Image-based computational models were constructed based on patient-specific lead locations and on individual stimulation settings to visualize the active contact locations across patients and to identify the structural networks and local fiber pathways modulated by DBS (Figure 5). The results highlighted the variability in applied stimulation across patients (Johnson et al., 2019). Structural connectivity of the site of stimulation and activation of specific local fiber pathways were predictive of improvement in tics and comorbid OCB (Johnson et al., 2020). The results could possibly be used to refine stimulation targets and to develop network-based approaches for DBS for TS in order to improve patient outcomes. Collectively, these analyses demonstrate the value of combining data across clinical centers in an effort to investigate DBS for less common indications.

### Predicting DBS Outcomes

Deep brain stimulation is an effective treatment for PD, but its efficacy depends heavily on selection of optimal stimulation parameters for each individual patient. DBS programming is frequently time consuming and burdensome for patients, caregivers, and clinicians. We recently conducted a multi-center study (NCT02474459) to test if the integration of the Mobile Application for PD DBS (MAP DBS), a clinical decision support system, into the DBS programming process could transform the care model by enabling home health nurses to effectively manage patients at home. We conducted two open-label, 1:1 randomized, controlled, clinical trials. The first trial, which was conducted at six expert DBS centers across the United States, compared 6 months of SOC to 6 months of MAP DBS-aided programming. The primary outcome was the total time spent on DBS programming over all clinical visits during the study period. In the second trial, we compared 6 months of SOC to 6 months of home health postoperative DBS management. The home health postoperative management was conducted by a home health nurse who chose DBS settings with the aid of the MAP DBS system. By design, the home health nurse had no prior experience providing DBS care. The primary outcome was the number of times each patient traveled to the movement disorders clinic during the study period. In both studies, the secondary outcomes were changes in the Unified Parkinson’s Disease Rating Scale (UPDRS) part III (motor score), the total UPDRS (sum of parts I through IV), the 39-question Parkinson’s disease rating scale (PDQ-39), the Multidimensional Caregiver Strain Index (MCSI), and levodopa equivalent daily doses (LEDD) between the baseline and six-month outcomes visit. We analyzed 72 (SOC = 37, MAP DBS = 35) patients in the first trial (Phase I). In the second trial (Phase II: home health nurse management) we analyzed 42 patients (SOC = 19, home health = 23). The study results are in submission but will help us to understand how tools like MAP DBS can be used to enhance the experience of non-expert home health nurses. Additionally, this data will be used to design and pilot a study of home health nurse driven telemedicine in DBS.

## Chronic Brain Sensing and Adaptive DBS

Seminal papers on adaptive neuromodulation and brain sensing have been primarily demonstrated in clinic or in postoperative settings (Pina-Fuentes et al., 2017). While the next generation device (i.e., Medtronic’s Summit RC + S) is being released, which includes chronic neural sensing, long-term home behavioral measurements may now be performed. These findings validate what has been reported in short perioperative settings, such as beta power alterations during DBS. Additionally, chronic sensing allows us to continually analyze pathological biomarkers, both subcortically and cortically, or brain states and how they respond to stimulation, aiding in the design of adaptive deep brain stimulation (aDBS) paradigms. Furthermore, as aDBS expands into real-world scenarios, the importance of defining individual biomarkers and the optimal site for sensing develops along with the utility of stereoelectroencephalography (sEEG) recordings before implantation of the DBS lead placement (Sanger et al., 2018; Shirvalkar et al., 2018). aDBS demonstrates promise in treating complex dynamics in signals (such as beta bursts) (Tinkhauser et al., 2017a, b; Deffains et al., 2018) or in disease states (such as pain or epilepsy).

However, if we want to achieve optimal aDBS outcomes, not only will electrode location and biomarker sensing be key, but also, the type of control algorithm used will ultimately affect the outcome and success of any aDBS paradigm. Previous control algorithms for PD have used a pre-specified threshold on beta (Little et al., 2013; Pina-Fuentes et al., 2017; Arlotti et al., 2018) or gamma (Swann et al., 2018) power, voltage linearly following beta power (Rosa et al., 2015), or dual threshold designs also on beta power (Velisar et al., 2019; Petrucci et al., 2020). Newer designs have focused on temporal dynamics of beta (Tinkhauser et al., 2017a). How complex must aDBS algorithms be to capture the dynamics of pathological biomarkers, especially as other indications arise like chronic pain? Overall, preliminary studies with chronic brain sensing will likely lead the way to a better understanding of neural circuitry under various medication and stimulation states and lead to the development of more sophisticated methods for targeting, individual neural biomarker or symptom identification profiles, and aDBS protocols across neurological disorders.

### Chronic Sensing and Closed-Loop Approaches in Parkinson’s

**FIGURE 6:**
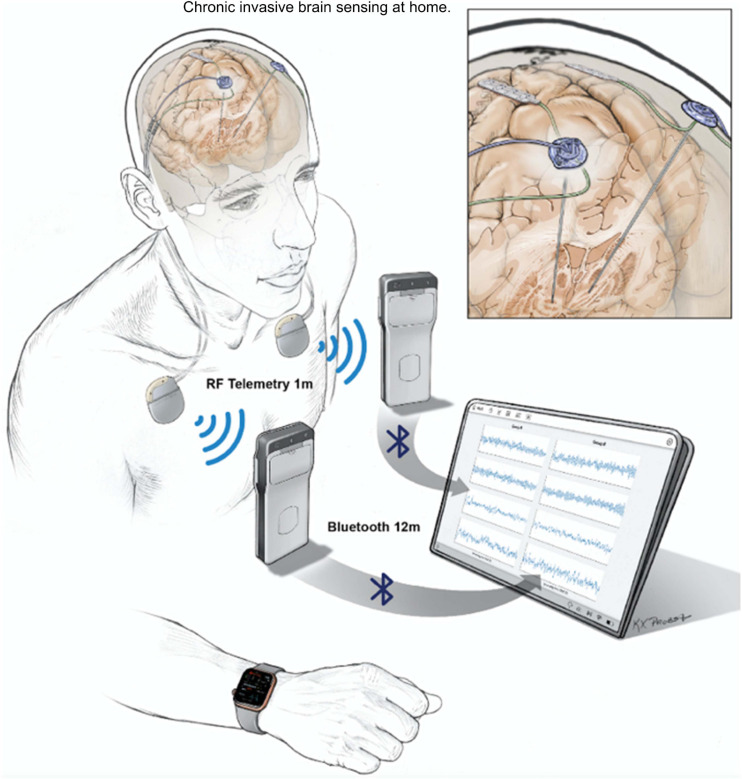
UCSF protocol for data streaming from Summit RC + S. Quadripolar leads were placed bilaterally into the subthalamic nuclei and over the motor cortex. Leads were connected to the ipsilateral Summit RC + S neural interfaces. Each RC + S device wirelessly communicates with a pocket-sized relay device, usually worn on the patient. The relay devices transmit by Bluetooth to a single small Windows-based tablet at a distance of up to 12 m, allowing sensing of local field potentials from up to four bipolar electrode pairs for up to 30 h per device, before recharge is needed. Data from a wristwatch-style actigraphy monitor (Parkinson’s Kinetograph, Global Kinetics) are synchronized off-line with neural recordings to facilitate brain-behavior correlations.

Invasive neural recordings in humans have shown promise for understanding physiological signatures or “biomarkers” of specific motor and non-motor signs of PD. Until recently, most recordings were performed for short durations from externalized brain leads in hospital settings. The availability of bidirectional (sense and stimulate) neural interfaces has launched a new approach: chronic invasive brain sensing at home. This approach offers many advantages over brief recordings: validation in the “real world” of biomarkers identified at rest with externalized leads in defined medication states, identification of “personalized” biomarkers based on chronic recordings over many exacerbations and remissions of a specific sign or symptom within a single subject, understanding effects of chronic DBS on neural circuits, and implementation of aDBS. Here, we highlight several uses of chronic brain recordings in PD at UCSF. Using an investigational first-generation bidirectional interface, the Activa PC + S (Medtronic) in four patients, we identified prefrontal cortical beta band activity as a possible signature of anxiety and depression. More recently we have used a second-generation bidirectional interface, Summit RC + S (Medtronic). This device has the capability for high volume wireless data streaming at home over many hours, improved signal to noise ratios, and better management of stimulation artifacts as compared to its precursor device. Five PD patients streamed bilateral 4-channel motor cortex and basal ganglia field potentials at home for over 2,600 h. Recordings were paired with wearable monitors for the neural decoding of motor fluctuations at home (Figure 6). We validated personalized neural biomarkers during normal daily activities. We examined the effects of chronic DBS and of sleep on these biomarkers and implemented aDBS at home, using both cortical and subthalamic signals to track motor fluctuations.

### Closed-Loop Modulation and Brain State Tracking for Epilepsy

Electrical brain stimulation (EBS) is an effective therapy for neurological and psychiatric diseases. Currently available systems, however, do not provide a bi-directional interface suitable for ambulatory biomarker tracking, patient reporting, or adaptive therapy. While regulatory challenges exist, integrating EBS implants with off-the-body computing devices, like a smart phone, can enable tracking, analyzing, and modulating brain activity in ambulatory subjects while also providing real-time behavioral data via phones and wearable sensors. The bi-directional interface between ambulatory patients, their brain activity, as well as the local and distributed computing environments creates a powerful platform for therapy optimization and neuroscience discovery.

**FIGURE 7:**
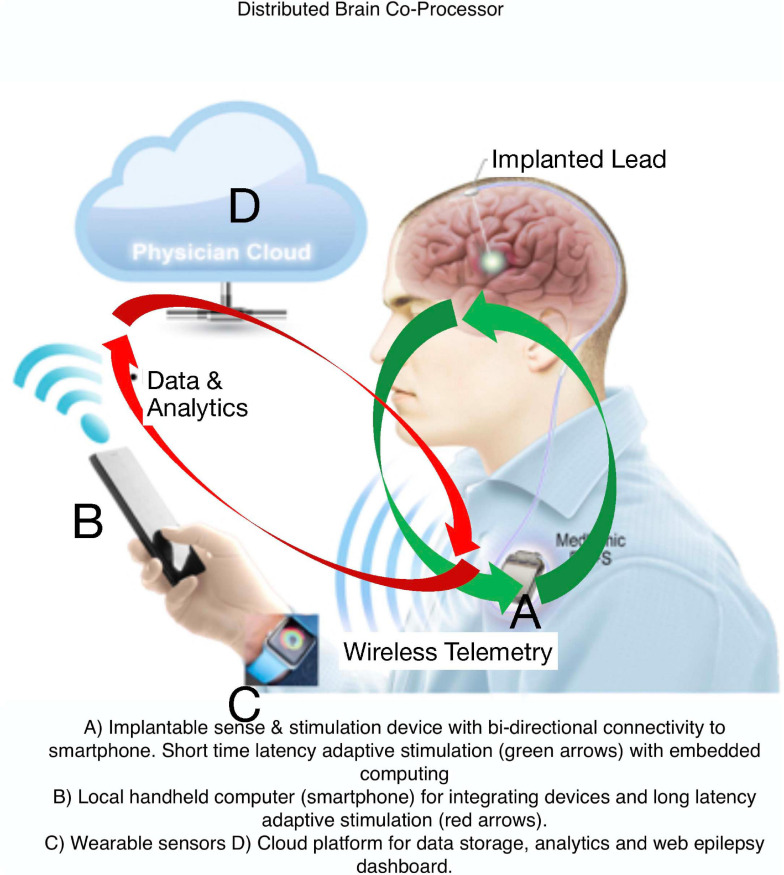
Digital Platform for Neurological and Psychiatric Disease. Integration of brain implants, smart phones, wearable sensors, and computing environments that provide data analytics synchronized with biomarker or user triggered interactions that can enable new therapy paradigms.

Current FDA approved EBS devices for epilepsy do not utilize adaptive therapy, take years for therapy optimization, and do not track seizures or treat common comorbidities like mood, sleep, and cognition. Here, we describe a Digital Epilepsy Management System for drug resistant epilepsy that integrates a brain stimulation and sensing implant with off-the-body devices and cloud computing enabled ambulatory tracking of seizures, biomarkers, behavior, sleep, cognition, and mood that can all possibly drive adaptive therapy (Figure 7).

Pre-clinical work at the Mayo Clinic was completed in research and pet canines with epilepsy using an investigational Medtronic Summit RC + S (bilateral hippocampus and anterior nucleus of the thalamus) sensing and stimulation implantable device integrated with a tablet computer and cloud-based system for streaming data acquisition from the brain and wearable sensors, patient and device triggered tablet annotations, data analytics, and visualization.

The system is currently deployed in a person with epilepsy and 2 pet dogs with epilepsy living with their owners. We have demonstrated automated seizure catalogs, interictal biomarker tracking, sleep staging, and patient mood and cognition testing in the naturalistic settings. The Digital Epilepsy Management System provides an interactive interface between patient and physicians and should be useful for optimizing adaptive EBS therapy in patients with epilepsy.

### Closed-Loop DBS for Refractory Chronic Pain

A diverse array of chronic pain syndromes is refractory to almost all treatment modalities; however, they involve pathological activity in similar brain regions. This finding suggests therapeutic potential for DBS to treat pain, but despite early promise, long-term efficacy is lacking. Prior DBS approaches have been limited in anatomical reach, targeting brain regions underlying only single dimensions of pain (such as somatosensation). Further, DBS therapy has been bluntly applied in an open-loop, continuous fashion without regard to underlying physiology. As a result of these shortcomings, DBS for pain is frequently ineffective or shows diminished effect over time. DBS could be significantly improved by seeking individually optimized brain targets or by using neural biomarkers of pain to selectively control stimulation when it is needed (closed-loop DBS). This type of approach may help avert tolerance and may provide prolonged pain relief.

Using personalized neural signatures of acute and chronic pain states over long-time scales (weeks to months), we at UCSF are developing a closed-loop DBS technology to treat chronic pain. In our first cohort of four subjects with chronic pain, we have implanted electrodes in the anterior cingulate and orbitofrontal cortices. Using machine learning methods, we have successfully decoded high versus low pain states and identified personalized biomarkers of clinically relevant chronic pain states. The stimulation of anterior cingulate cortex (ACC) and orbital frontal cortex (OFC) has been variably analgesic with inconsistent results over time. In a newer study, we are using sEEG to perform a temporary stimulation trial of multiple brain targets that underlie the somatosensory, affective, and cognitive dimensions of pain. Based on this trial period, we can identify personalized brain targets for each subject to maximize both stimulation-induced pain relief and pain biomarker detection. These pain biomarkers can then be used as a next step and be embedded into closed-loop DBS control algorithms to provide long term pain relief.

## Impulsivity and Neuropsychiatric Aspects of DBS

As DBS has proven successful in well-selected patients for the treatment of movement disorders, such as PD and essential tremor, its indications are expanding to include intractable or severe neuropsychiatric conditions such as post-traumatic stress disorder (PTSD; Larkin et al., 2020), OCD (Goodman et al., 2020), and impulsive behavior (Wu et al., 2020). As DBS expands into neuropsychiatry, several questions and opportunities for development arise. These include optimal targets or targeting, either anatomical or functional-based, individualistic biomarkers of pathology, which can aid in the development of aDBS paradigms, identifying circuitries involved in psychiatric disorders, patient selection and ideal stimulation paradigms (i.e., open- vs. closed-loop strategy).

### Intracranial Neurophysiological Biomarkers of Hypervigilance and Fear in Humans

The ability to detect and subsequently remember threats is critical for survival. However, extreme, or life-threatening situations, can produce long-standing changes in fear-processing circuitry and these can lead to anxiety disorders such as PTSD. Through a collaboration between Jean-Philippe Langevin, members of the Suthana laboratory, and colleagues at the Veteran’s Administration Greater Los Angeles Healthcare System, we were able to record intracranial electroencephalographic (iEEG) activity in veteran participants with implanted electrodes placed within amygdala, hippocampal, and prefrontal regions. Participants included those with an implanted RNS^®^ System (NeuroPace, Inc.), sEEG electrodes for seizure evaluation, or intra-operative recording electrodes implanted prior to DBS placement in a patient with PTSD. A subset of the participants was diagnosed with PTSD and/or generalized anxiety disorder (GAD). Results yielded low and high frequency oscillatory biomarkers that were and will be used to trigger responsive neurostimulation in PTSD patients as part of an ongoing NIH UH3 funded clinical trial. Future studies will focus on characterizing amygdala-hippocampal-prefrontal circuit mechanisms underlying fear-related memory and improve the ecological validity of laboratory-based tasks using virtual reality, simultaneous physiological (e.g., heart rate, skin conductance, and pupillometry) and iEEG activity combined with intracranial electrical stimulation [for methods see (Topalovic et al., 2020)].

### Development of Adaptive DBS for OCD

Ventral striatum (VS) DBS for treatment of intractable OCD benefits approximately 50–60% of cases, leaving room for improvement in both clinical outcomes and reduction of DBS-induced behavioral side effects, notably hypomania. An adaptive DBS (aDBS) system may improve efficacy of DBS for OCD by facilitating the titration of stimulation parameters in response to neural biomarkers of hypomania and in response to OCD-related distress. In an NIH UH3 (NS100549; PI Goodman) for aDBS in OCD, five participants underwent DBS surgery; two with the Medtronic Activa PC + S, and three with the Medtronic Summit RC + S. Participants returned to clinic for DBS programming and to conduct neural recordings [local field potentials (LFPs) and scalp EEG], video and heart rate monitoring, as well as behavioral tasks, all measured on a bi-weekly to monthly basis. Early evidence from one participant suggested an increase in the left vs delta-band power over a timeline of weeks since the DBS ON condition. This increase in power preceded symptom improvement, which occurred after an increase in pulse width from 90 to 120 μs that elicited a mirthful response. In addition to in-clinic data collection, we captured data in the participants’ home environments. The Summit RC + S device enabled streaming of neural data at home during natural fluctuations in OCD symptom intensity and hypomania, as well as during exposure response therapy. There were 228 h of neural data streamed from one Summit RC + S participant during a range of behavioral states and tasks. The majority of LFP data was collected during active DBS therapy. To better understand underlying neural activity, we developed a novel stimulation artifact removal paradigm, termed Period-based Artifact Reconstruction and Removal Method (PARRM). PARRM is applicable to various neurostimulation paradigms beyond DBS, is superior in signal recovery, low complexity, and requires minimal onboard storage. Next, we plan to examine behavioral and neural data collected across various behavioral states to identify biomarkers that can be deployed in an effort to enable aDBS for OCD.

### Closing the Loop on Impulsivity With Deep Responsive Neurostimulation: Past, Present, and Future of BITES

Loss of control (LOC) is a pervasive feature of eating disorders and contributes significantly to the epidemic of obesity. Responsive DBS that is guided by low-frequency changes in the nucleus accumbens (NAc), was previously observed to block binge-eating behavior in mice (Halpern et al., 2013). Following novel preclinical work and a human case study which demonstrated an association between the delta-band (1–4 Hz) and reward anticipation, an Investigational Device Exemption for a first-in-human trial was approved by the US FDA (Brain Intervention Therapy for Eating Suppression, the BITES study). BITES is a single site (Stanford University), early feasibility trial with a randomized, single-blinded, staggered-onset design. Six participants will undergo bilateral DBS of the NAc for LOC eating using the RNS^®^ System (NeuroPace, Inc.). Eligible participants must have treatment-refractory obesity with a body mass index (BMI) 40–60 kg/m^2^.

There are three participants currently enrolled. Electrophysiological signals of LOC will be characterized in humans using ambulatory recording capabilities and controlled, in-clinic behavioral tasks. We have developed novel behavioral tasks and we will utilize virtual reality and eye-tracking to capture anticipatory signals for LOC eating during intraoperative testing and in the laboratory. Using eye-tracking and remote telemetry communication, we captured real-time electrophysiological signals during naturalistic eating behaviors in the clinic. We assess LOC eating in the clinic by introducing participants to a validated multi-item buffet task where they are given a standard breakfast and lunch (500 kcal/meal), and following a brief LOC priming period with mood provocation, participants are presented a buffet of preferred, high fat-caloric food (∼5,000 kcal). Further, we utilize ambulatory data collection via magnet swiping which is paired with ecological momentary surveys, food diaries, and wearables to capture real-world LOC cravings and eating episodes. Initial piloting of these tasks and assessments were used for feasibility in the initial pilot study and analysis for a LOC-responsive biomarker study. Collectively, these preliminary results demonstrate the usefulness of long-term, objective neural recordings in naturalistic environments and the potential of individualized biomarkers of pathology or symptoms for the potential successful employment of aDBS.

## Emerging Techniques for DBS

New techniques for the application of DBS have emerged with the advent of imaging, which has resulted in a paradigm shift toward targeted modulation of a particular network (Gonzalez-Escamilla et al., 2019, 2020; Horn et al., 2019). Another emerging and as yet unresolved area is how beta oscillatory activity in the basal ganglia is affected by DBS and how this is associated with symptom improvement (Lofredi et al., 2019; Petersson et al., 2020). Studies demonstrating suppression of beta activity by DBS revealed that this was associated with amelioration of symptoms, and also with an attenuation of this effect after continuous stimulation (Chen Y. et al., 2020). This section summarizes the latest information on device technology, structural/functional aspects, and biomarkers for improved DBS programming.

### Update on Emerging Technologies and Deep Brain Stimulation

Initially, DBS was thought to mimic the effect of lesioning through neuromodulation of neuronal activity within the area surrounding the stimulating electrode. However, current physiological concepts imply, that DBS has multiple, time-dependent effects on the cellular, local neuronal circuitry and also at the large-scale network level. These changes may influence the dysfunctional activity within symptom specific neural circuits. Axons originating or terminating within the stimulation volume or bypassing it are the key elements potentially mediating multiple clinical responses (Kuhn and Volkmann, 2017; Lozano et al., 2019). Therefore, ideal neurostimulation technology.

• should be flexible in stimulating only a small volume of interest,
• should preferentially stimulate axons mediating benefit,
• should avoid stimulation of axons or other excitable elements resulting in adverse effects,
• should eliminate the neuronal signal mediating a network dysfunction (e.g., oscillopathy),
• should not interfere with normal (physiological) network function.

Recent methodological advances addressing these needs include segmented electrode designs and an expanded pulse parameter space (e.g., shorter pulse durations, anodic stimulation) for more precise delineation of the stimulation volume and for selective stimulation of particular fiber pathways of interest. Another advance includes sensing capability using either brain signals or peripheral kinematic sensors to adapt stimulation to fluctuating symptom severity and to the underlying dynamics of neural circuits. Finally, future advances include the rapidly advancing field of digital innovations for clinical response prediction which may inform and substantially shorten programming times for DBS.

Several open source and commercial software have facilitated visualization of the volume of tissue activated (VTA) based on axon cable models in patient specific anatomical models (derived from MRI and CT imaging). Aggregated data from large patient cohorts have facilitated the creation of probabilistic maps of clinical responses, which in turn can be used to train machine learning algorithms for predicting a clinical (individual) response with a given electrode location and a particular stimulation setting (Reich et al., 2019). We hope, that resulting “expert systems” will help to better manage DBS patients in a more reliable and consistent way across centers and will reduce the need for high-level individual expertise in DBS programming. A clinical trial (site: University of Würzburg, Germany) comparing machine based and best clinical programming in dystonia has recently received funding from the German Ministry of Research and Technology and will be initiated in 2021 (DIPS: Dystonia Image-based Programming of Stimulation: A prospective, randomized, double-blind crossover trial).

### Structural and Functional Network Characterization for Prediction of DBS Patients

Despite the vital role of brain network studies to predict disease trajectories in patients with movement disorders, their analysis and modeling are often difficult to interpret due to complexity, uniqueness, test-retest issues and group or single subject validity of the data. Of distinct importance for the comprehension of the analytical framework is to note, that network interactions occur at specific spatial locations within regions (space) over distinct time dimensions (state). With this in mind, we hypothesize that brain networks have instantaneous state and space properties at each level. Proper identification and association of features (within and between brain regions) from these critical variables at different time scales can be modeled by newly proposed computational approaches (Gonzalez-Escamilla et al., 2020; Muthuraman et al., 2020) that can then have the potential to predict individual responses to DBS.

**FIGURE 8:**
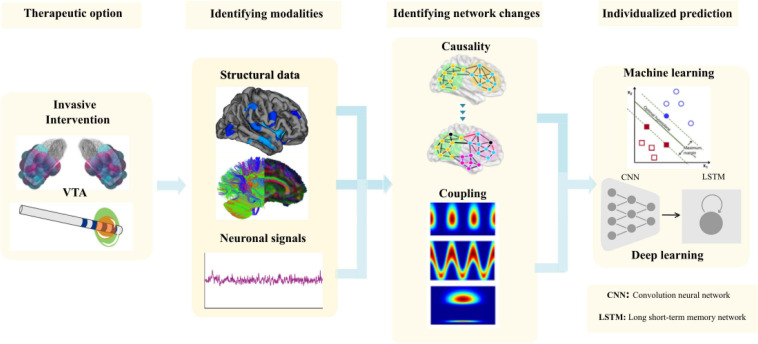
The methodological framework to entangle network proxies for prediction of the outcome of the DBS patients from volume of tissue activated (VTA) modeling to identifying the correct modalities to identify the network changes and use it for individual prediction using matching learning algorithms.

In a recent review, we comprehensively describe the causal interrogations and modulations of network states using neuroimaging and electrophysiology (Gonzalez-Escamilla et al., 2020). Using structural magnetic resonance imaging (sMRI), we were further able to show in the pre-operative MRI the cortical thickness (CT) in the frontal lobe predicted the clinical improvement after STN-DBS (Muthuraman et al., 2017) and cortical atrophy in sensorimotor areas in dystonia patients (Gonzalez-Escamilla et al., 2019). In the same direction, frontal lobe network proxies can predict postoperative clinical response to STN-DBS using diffusion tensor imaging (DTI; Koirala et al., 2018). By using functional state recordings and analyses from electroencephalography (EEG) and electromyography (EMG) we were able to show the topography of oscillatory coherent sources in the cerebellum and sensory-motor cortex could robustly separate patients with different tremor syndromes and act as variables for closed-loop approaches (Muthuraman et al., 2018). Moreover, in advanced network analyses using a similar analytical framework with high-density EEG we described cross frequency coupling as a marker for clinically effective DBS of the STN-DBS, that modifies fine-tuned gamma oscillations for the optimal clinical response (Muthuraman et al., 2020). After identifying these network proxies (Figure 8), the aim is for rapid translation of scientific knowledge to clinical practice. There is a clear need for testing of this proposed state- and space framework in the clinical setting.

### Local Field Potentials as Biomarkers for DBS Control and Programming

Over the last decade, we and others have used the access to deep brain nuclei in patients undergoing DBS not only for treatment of motor symptoms, but also to record neuronal activity in order to understand the underlying pathophysiology of movement disorders. We could show that the temporal pattern of neuronal output is highly important to understanding network disorders. The oscillatory activity pattern is different in bradykinetic and hyperkinetic disorders, i.e., dynamic changes in the network related to a specific motor state of the patient. Best known is the increased subthalamic oscillatory beta (13–35 Hz) band activity which has been shown to be a potential electrophysiological signature of bradykinetic motor signs in patients with PD (Silberstein et al., 2003). Dopaminergic medication has been associated with a decrease of this pathologically enhanced activity, specifically in the low beta sub-band (13–20 Hz) (Kuhn et al., 2006, 2008).

In several studies, a significant correlation between parkinsonian symptom severity and beta synchronization has been reported (Kuhn et al., 2006, 2008), even months after neurostimulator implantation (Neumann et al., 2017). Moreover, beta band activity has been shown to be suppressed by neuromodulation (Kuhn et al., 2008; Eusebio et al., 2011). Research has focused on the improvement of DBS and there has been movement toward more patient-tailored, adaptive stimulation (aDBS). The idea of aDBS has been to switch stimulation ON/OFF or to modulate the stimulation amplitude in response to the real-time analysis of a potential biomarker, e.g., beta band activity in PD (Little et al., 2013). A recent technical development has facilitated chronic sensing using the PERCEPT (Medtronic) pulse generator. This new technical advancement will help to define the biomarker as a feedback signal for future adaptive DBS.

Our first experience with PERCEPT has revealed that a stepwise increase of stimulation amplitude was mirrored in the sequential decrease of beta oscillatory activity, which occurred in parallel with the improvement in bradykinesia. Mean beta band (13–20 Hz) activity correlated significantly with the UPDRS scores during DBS. Further studies using chronic sensing will likely reveal circadian fluctuations in oscillatory patterns. This will also likely be useful for application to aDBS in real life situations. This finding may also be important to future clinical development.

## Neurotechnology and Neuroengineering in DBS Research

This section describes the advances in the field of neurotechnology as applicable to DBS research. This includes real-time neural recordings, remote DBS programming, optimization of electrode configurations, and model-based algorithms, which can all be developed to further incorporate physiological signals in the optimization process. Finally, we address issues surrounding chronic implantation and utilization of neural micro-devices, which have the potential to provide sensory information feedback, and how to mitigate these issues.

### Real Time Recording of EEG and ECG Using DBS Electrodes

With sensing-enabled deep brain stimulators, chronically monitoring neural activities in the deep brain has become a reality. This capability could play a key role in clinical neuroscience and neuromodulation technology. We developed a DBS system with the capability of chronic recording (Qian et al., 2016). We investigated artifact removal methodologies (Qian et al., 2017a, b) and we built a software platform to improve signal recording and signal processing. Based on this technology, we conducted longitudinal clinical recordings to observe chronic LFPs and the effects of DBS on neuromodulation (Qian et al., 2016). The results have revealed that there may be a chronic change in the beta suppression of DBS in the subthalamic nucleus of PD patients.

**FIGURE 9:**
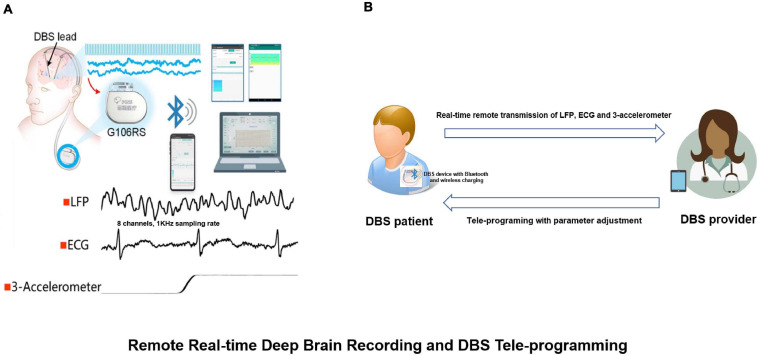
Remote real-time deep brain recording and DBS tele-programming. **(A)** The G106RS system, a sensing-enabled DBS with Bluetooth connection, can monitor deep brain rhythms remotely. Specifically, it was capable of transmitting up to eight channels of local field potentials (LFPs) with 1,000 Hz sampling rate, one channel of electrocardiogram (ECG) and 3-D accelerometer signals; **(B)** DBS patient equipped with G106RS device with Bluetooth connection and wireless charging. A tele-programming DBS system can remotely adjust parameters via Bluetooth technology by the provider. The LFP, ECG and 3-D accelerometer signals can be transmitted remotely from the G106RS device to a data receiver accessed by the provider.

In addition to this change we also observed combinations of alpha, beta and gamma bands which could be used as chronic biomarkers for the classification of different sleep stages (Chen et al., 2019). The results have guided the development of a closed-loop DBS approach. Recently, this sensing-enabled device has been improved by employing Bluetooth communication, facilitating the potential for the application of implantable brain-machine interfaces. The latest advance has been the first Bluetooth DBS device which was implanted in China. The device could directly connect to a mobile device by Bluetooth and was capable of transmitting up to eight channels of LFPs, one channel of ECG as well as 3-D acceleration signals (Figure 9).

### Remote DBS Programming During COVID-19 Pandemic

In the past five years, remote programming technology has been widely expanding in large medical centers specifically in an effort to improve access, and particularly for those residing in remote locations. At the beginning of the COVID-19 pandemic, many patients were secluded from standard medical services due to social distancing, quarantine, and lockdowns. For patients with PD, efficient programming could be safely achieved by a remote tele-programming system. We have developed a DBS programming technology using a Bluetooth communication interface. We reported the application of the device during the pandemic, particularly for PD patients with freezing of gait (FOG) who were able to be programmed with a complex variable frequency stimulation (VFS; Jia et al., 2018) paradigm (Zhang et al., 2020). This technology could potentially be shared among multiple medical centers when paired with the implementation of adequate privacy protections.

Our remote programming system provides a promising approach for the control of a wide range of implantable medical devices. We anticipate that telemedicine for remote DBS programming will be an important trend in future DBS management.

### Model-Based Algorithms for Optimizing DBS Therapy

Computational models of DBS have provided significant insight into the regions and pathways involved in treatment and side effect induction with DBS therapy. To date much of the research has focused on retrospective analysis in which previously collected clinical outcomes are matched with model predictions of the regions or pathways activated by clinically optimized or suboptimal stimulation settings that may or may not induce side effects. As we continue to pursue these retrospective studies, the knowledge gained provides an opportunity to build data-driven algorithms for identifying therapeutic electrode configurations prospectively and on an individual subject basis.

Several targeting algorithms have been developed in recent years including neural network classification based on VTA morphologies (Chaturvedi et al., 2013), convex optimization for targeting several (Xiao et al., 2016), or a broader range (Anderson et al., 2018) of axonal pathways, orientation-selective stimulation (Lehto et al., 2017; Slopsema et al., 2018, 2020) and particle swarm optimization that can incorporate single (Pena et al., 2017) or multiple (Pena et al., 2018) objective functions. While these studies have focused primarily on optimizing electrode configurations, including monopolar and multipolar stimulation, and stimulation amplitude, model-based algorithms have also shown utility for optimizing the pattern of stimulation (Brocker et al., 2017; Cassar et al., 2017) and the shape and size of DBS (Howell et al., 2015) electrodes (Teplitzky et al., 2016).

**FIGURE 10:**
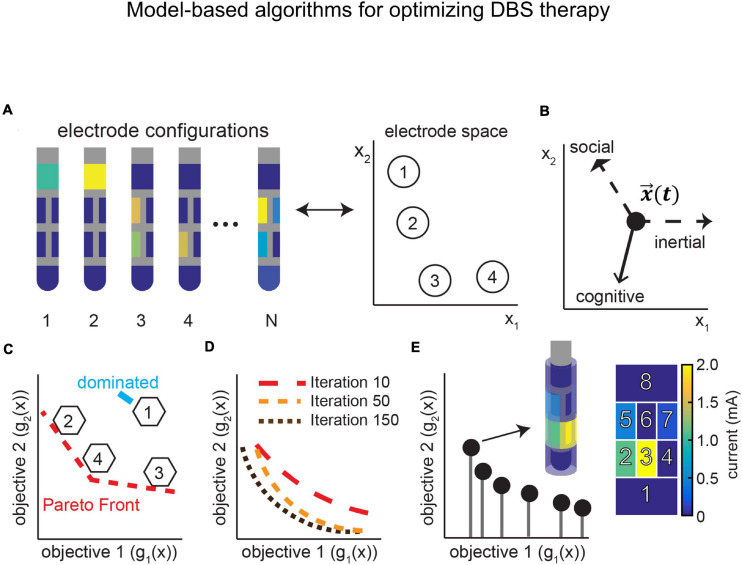
Multi-objective particle swarm optimization algorithm for determining DBS parameter sets that more selectively active one or more axonal pathways adjacent to a DBS lead. **(A)** Multiple particles explore electrode configurations and stimulation amplitudes, and are guided by panel **(B)**, an inertial, cognitive, and social component amongst the N particles. **(C–E)** Particles are mapped onto a multi-objective space that describes the goal of activating one or more pathways over other pathways within the brain. Through an iterative process, non-dominated particles are tracked to create a Pareto front with particles corresponding to optimized electrode configurations. Reproduced with permission from Pena et al. (2018).

Model-based algorithms can also be extended to incorporate physiological signals in the optimization process. Examples of these approaches include adaptive closed-loop strategies that integrate a response surface model to intelligently guide and update stimulation parameters. One recent example is the development of Bayesian adaptive dual control that balances exploitation of DBS settings that are known to be therapeutic with the exploration of settings that may yield a better outcome (Grado et al., 2018; Figure 10). As telehealth becomes more mainstream for DBS programming and in cases in which the clinical effects of DBS have long wash-in and wash-out time constants, model-based optimization algorithms are poised to make a significant future impact.

### Engineering the Neuronal Response to Electrical Microstimulation

The loss of sensorimotor function has devastating consequences on quality of life. One approach to restoring lost sensorimotor abilities is to supply patients with implants that provide a direct interface with the central nervous system. For an amputee or tetraplegic patient, this interfacing could allow a patient’s desired limb movement to be executed by a prosthetic limb, and to convey to the patient, sensory information about the consequences of these movements. Highly sophisticated robotic limbs have been developed, as have algorithms to decode motor commands from the brain. However, somatosensory feedback is critically important in activities of daily living. Furthermore, touch is important in emotional communication and in embodiment of our limbs. Without touch, the dexterity of the prostheses will be limited, as will the degree to which they are incorporated into the self-image. Given the importance of touch, upper limb neuroprostheses may not be clinically viable until these devices provide informative tactile feedback.

Direct interfacing of micro-devices with the brain has the potential to provide sensory information feedback. However, chronic implantation and utilization of neural micro-devices can result in a reactive tissue response that both functionally isolates the device from the tissue as well as triggers neuronal apoptosis or migration. The goal of our research is to understand and to mitigate this limited functionality. Our research seeks to determine the interdependent effects of device design, electrophysiological recording, electrical stimulation, and the reactive tissue response on the efficacy of neural interfaces. We: (1) conduct psychophysical experiments using multi-channel cortical implants in the cortex, (2) collect longitudinal electrochemical and electrophysiological recordings, (3) investigate mitigation strategies, and (4) use advanced histological approaches to evaluate the device-tissue interface. Our lab studies these various approaches and their implications for reliable chronic neural interfacing via micro-devices. We expect that these data will enable further neuroprosthetic development for many neural interfaces’ potential applications.

## Summary and Conclusion

The Eighth Annual DBS Think Tank meeting provided scientific insight into the most current commercially available technologies and also facilitated important dialogue as to how clinical outcomes may be influenced The topics included (1) closed-loop and adaptive DBS for the treatment of multiple emerging indications, (2) improved imaging techniques which could expand our understanding of brain circuitry and improve DBS outcomes by offering more personalized targeting, (3) optogenetics to elucidate the fundamental roles of various cell types in the neurobiology of disease and could lead to a better understanding of pathological brain circuitries, and (4) the use of chronic neural recordings to define symptom-specific, individualized biomarkers. The Think Tank also addressed a multitude of emerging ethical issues arising from research and from the application of these aforementioned technologies, especially when DBS is successfully applied for off-label uses. We discussed the ethical implications of post-trial management. Furthermore, attendees of the DBS Think Tank completed a questionnaire and 178 participants responded. The participants were primarily scientists or clinicians at academic institutions/universities. The weighted-mean experience in the field of neurotechnology of the participants was 12.3 years. Within the last year, DBS for essential tremor and PD remain at the slope of enlightenment, with mean scores of 5.38 and 5.36, respectively. Additionally, cochlear implants have joined the slope of enlightenment this year. Optogenetics for clinical neural interfaces remains as a technology trigger. Several DBS indications (PTSD, obesity, traumatic brain injury, addition, Alzheimer’s) have moved from technology trigger to peak of inflated expectations, corresponding to the expanding research and clinical trials. Results indicated that some uses and techniques of DBS remained on the trough of disillusionment (intraoperative physiology, imaging post DBS implant, DBS for epilepsy, DBS for Tourette) or moved to the trough of disillusionment (DBS for OCD, DBS for chronic pain, closed-loop DBS).

These proceedings present the latest advances in the field of neuromodulation and emerging challenges that will require international collaboration to more rapidly advance DBS therapy.

## Data Availability Statement

The original contributions presented in the study are included in the article/supplementary material, further inquiries can be directed to the corresponding author.

## Ethics Statement

The studies involving human participants were reviewed and approved by the individual academic institutions. All participants provided their written informed consent prior to participation in the studies. The animal study was reviewed and approved by individual institutional animal welfare committees at the respective academic institutions.

## Author Contributions

All authors fulfilled authorship criteria by substantial contributions to the conception of the work, providing data for the work, revisiting it critically for important intellectual content, approving the final version, and agreeing to be accountable for all aspects of the work in ensuring that questions related to the accuracy or integrity of any part of the work are appropriately investigated and resolved.

## Conflict of Interest

SG and MM were employed by the companies Medtronic and Boston Scientific Neuromodulation, respectively. Research devices for Dr. Goodman’s NIH funded study were donated by Medtronic. The remaining authors declare that the research was conducted in the absence of any commercial or financial relationships that could be construed as a potential conflict of interest.

## Footnotes

1
https://www.cardiovascular.abbott/us/en/patients/living-with-your-device/arrhythmias/remote-monitoring.html

2
https://www.bostonscientific.com/content/gwc/en-US/products/remote-patient-monitoring/heart-connect-system.html

3
https://anzctr.org.au/Trial/Registration/TrialReview.aspx?ACTRN=12619001660178
