# Corrigendum: Proceedings of the Eighth Annual Deep Brain Stimulation Think Tank: Advances in Optogenetics, Ethical Issues Affecting DBS Research, Neuromodulatory Approaches for Depression, Adaptive Neurostimulation, and Emerging DBS Technologies

**DOI:** 10.3389/fnhum.2021.765150

**Published:** 2021-10-01

**Authors:** Vinata Vedam-Mai, Karl Deisseroth, James Giordano, Gabriel Lazaro-Munoz, Winston Chiong, Nanthia Suthana, Jean-Philippe Langevin, Jay Gill, Wayne Goodman, Nicole R. Provenza, Casey H. Halpern, Rajat S. Shivacharan, Tricia N. Cunningham, Sameer A. Sheth, Nader Pouratian, Katherine W. Scangos, Helen S. Mayberg, Andreas Horn, Kara A. Johnson, Christopher R. Butson, Ro'ee Gilron, Coralie de Hemptinne, Robert Wilt, Maria Yaroshinsky, Simon Little, Philip Starr, Greg Worrell, Prasad Shirvalkar, Edward Chang, Jens Volkmann, Muthuraman Muthuraman, Sergiu Groppa, Andrea A. Kühn, Luming Li, Matthew Johnson, Kevin J. Otto, Robert Raike, Steve Goetz, Chengyuan Wu, Peter Silburn, Binith Cheeran, Yagna J. Pathak, Mahsa Malekmohammadi, Aysegul Gunduz, Joshua K. Wong, Stephanie Cernera, Wei Hu, Aparna Wagle Shukla, Adolfo Ramirez-Zamora, Wissam Deeb, Addie Patterson, Kelly D. Foote, Michael S. Okun

**Affiliations:** ^1^Norman Fixel Institute for Neurological Diseases and the Program for Movement Disorders and Neurorestoration, Department of Neurology, University of Florida, Gainesville, FL, United States; ^2^Department of Bioengineering, Stanford University, Stanford, CA, United States; ^3^Department of Psychiatry and Behavioral Sciences, Stanford University, Stanford, CA, United States; ^4^Department of Neurology and Neuroethics Studies Program, Georgetown University Medical Center, Washington, DC, United States; ^5^Center for Medical Ethics and Health Policy, Baylor College of Medicine, Houston, TX, United States; ^6^Weill Institute for Neurosciences, Memory and Aging Center, University of California, San Francisco, San Francisco, CA, United States; ^7^Department of Neurosurgery, David Geffen School of Medicine and Semel Institute for Neuroscience and Human Behavior, University of California, Los Angeles, Los Angeles, CA, United States; ^8^Department of Psychiatry and Biobehavioral Sciences, Semel Institute for Neuroscience and Human Behavior, University of California, Los Angeles, Los Angeles, CA, United States; ^9^Department of Psychology, University of California, Los Angeles, Los Angeles, CA, United States; ^10^Department of Bioengineering, University of California, Los Angeles, Los Angeles, CA, United States; ^11^Neurosurgery Service, Department of Veterans Affairs Greater Los Angeles Healthcare System, Los Angeles, CA, United States; ^12^Menninger Department of Psychiatry and Behavioral Sciences, Baylor College of Medicine, Houston, TX, United States; ^13^School of Engineering, Brown University, Providence, RI, United States; ^14^Department of Neurosurgery, Stanford University Medical Center, Stanford, CA, United States; ^15^Department of Neurological Surgery, Baylor College of Medicine, Houston, TX, United States; ^16^Department of Psychiatry, University of California, San Francisco, San Francisco, CA, United States; ^17^Department of Neurology and Department of Neurosurgery, Icahn School of Medicine at Mount Sinai, New York, NY, United States; ^18^Movement Disorders & Neuromodulation Unit, Department for Neurology, Charité – University Medicine Berlin, Berlin, Germany; ^19^Department of Biomedical Engineering, University of Utah, Salt Lake City, UT, United States; ^20^Scientific Computing and Imaging Institute, University of Utah, Salt Lake City, UT, United States; ^21^Department of Neurological Surgery, Kavli Institute for Fundamental Neuroscience, University of California, San Francisco, San Francisco, CA, United States; ^22^Department of Neurology, Mayo Clinic, Rochester, MN, United States; ^23^Department of Anesthesiology (Pain Management) and Neurology, University of California, San Francisco, San Francisco, CA, United States; ^24^Neurologischen Klinik Universitätsklinikum Würzburg, Würzburg, Germany; ^25^Section of Movement Disorders and Neurostimulation, Biomedical Statistics and Multimodal Signal Processing Unit, Department of Neurology, Focus Program Translational Neuroscience, University Medical Center of the Johannes Gutenberg-University Mainz, Mainz, Germany; ^26^Department of Neurology, Charité – Universitätsmedizin Berlin, Berlin, Germany; ^27^National Engineering Laboratory for Neuromodulation, School of Aerospace Engineering, Tsinghua University, Beijing, China; ^28^Department of Biomedical Engineering, University of Minnesota, Minneapolis, MN, United States; ^29^J. Crayton Pruitt Family Department of Biomedical Engineering, University of Florida, Gainesville, FL, United States; ^30^Restorative Therapies Group Implantables, Research and Core Technology, Medtronic, Minneapolis, MN, United States; ^31^Department of Neurological Surgery, Thomas Jefferson University Hospitals, Philadelphia, PA, United States; ^32^Asia Pacific Centre for Neuromodulation, Queensland Brain Institute, The University of Queensland, Brisbane, QLD, Australia; ^33^Neuromodulation Division, Abbott, Plano, TX, United States; ^34^Boston Scientific Neuromodulation, Valencia, CA, United States; ^35^Department of Neurology, University of Massachusetts, Worchester, MA, United States

**Keywords:** DBS (deep brain stimulation), neuroethics, optogenetics, novel hardware, adaptive DBS, neuroimaging

Wei Hu was not included as an author in the published article.

In the original article, we neglected to include the funders NPF and Tyler's Hope to Wei Hu.

In the original article, there was an error. A donation was omitted.

A correction has been made to the Conflict of Interest Statement, with the following sentence added:

“Research devices for Dr. Goodman's NIH funded study were donated by Medtronic.”

The authors apologize for this error and state that this does not change the scientific conclusions of the article in any way. The original article has been updated.

## Publisher's Note

All claims expressed in this article are solely those of the authors and do not necessarily represent those of their affiliated organizations, or those of the publisher, the editors and the reviewers. Any product that may be evaluated in this article, or claim that may be made by its manufacturer, is not guaranteed or endorsed by the publisher.

